# Innovative strategies in combating intervertebral disc degeneration: pathological mechanisms and biomaterial advancements

**DOI:** 10.3389/fbioe.2025.1643222

**Published:** 2025-08-14

**Authors:** Long Ma, Jizhou Pan, Jiankang Zhang, Fashun Liu

**Affiliations:** ^1^ Department of Pain Medicine, Sanya Central Hospital (The Third People’s Hospital of Hainan Province), Sanya, China; ^2^ Department of Emergency Surgery, Guizhou Provincial People’s Hospital, Guiyang, China

**Keywords:** intervertebral disc degeneration, oxidative stress, reactive oxygen Species, biomaterials, regenerative medicine

## Abstract

Intervertebral disc degeneration (IVDD) is a leading cause of chronic low back pain, significantly impacting the quality of life for millions of individuals worldwide. The onset of IVDD is associated with various factors such as age, lifestyle, and genetics, and its pathological mechanisms involve multidimensional interactions, including oxidative stress, inflammation, and extracellular matrix (ECM) metabolic disorders. During degeneration, there is a reduction in the number of nucleus pulposus cells (NPCs), resulting in an imbalance between the synthesis and degradation of the ECM, leading to changes in the disc’s morphology and biomechanical function, ultimately causing pain and mobility issues. As the global population ages, the incidence of IVDD continues to rise, necessitating the development of effective treatment strategies. Recent research into biomaterials, particularly hydrogels and stem cell technologies, has shown promise for disc regeneration, providing scaffolds to enhance cellular repair and facilitate drug delivery. This review comprehensively examines recent advancements in IVDD research, focusing on the pathological mechanisms and the potential application of biomaterials in treatment. Additionally, the integration of emerging technologies such as 3D printing and stem cell therapy represents a transformative approach in IVDD management. These findings open new avenues for targeted intervention strategies that address the underlying causes of IVDD, paving the way for improved clinical outcomes.

## 1 Introduction

IVDD is one of the primary causes of low back pain, significantly affecting the quality of life for millions of patients worldwide (Chenot et al., 2017). According to statistics from the GBD 2021 Low Back Pain Collaborators, approximately 619 million people suffer from low back pain, with IVDD playing a crucial role in this condition (Ferreira et al., 2023). IVDD may begin as early as adolescence, occurring earlier than degeneration in other musculoskeletal tissues; in most cases, this degeneration remains asymptomatic (Eksi et al., 2022). However, the incidence of IVDD continues to rise with age. For instance, the prevalence is 10% in men at age 50, increasing fivefold by age 70 (Kalichman et al., 2010). As the disease progresses, the height of the intervertebral disc (IVD) decreases, leading to alterations in spinal biomechanics. These changes expedite the degeneration of nearby spinal segments and other spinal structures, including ligaments and muscles. This process can ultimately lead to spinal canal stenosis and compression of neural tissues, resulting in pain and mobility issues (Colombini et al., 2008). This pain and mobility impairment not only lead to a decline in quality of life but also result in a long-term socioeconomic burden (Kos et al., 2019).

The IVD consists mainly of the nucleus pulposus (NP), annulus fibrosus (AF), and cartilaginous endplate (CEP). It functions to cushion spinal pressure and improve spinal mobility (Bowles and Setton, 2017). As a multifactorial disease, the occurrence and progression of IVDD are closely associated with various factors, including age, sex, lifestyle (such as smoking and obesity), genetics, trauma, and abnormal pathophysiological mechanical loading (Wang et al., 2023a). The pathological mechanisms of IVDD exhibit multidimensional, interactive characteristics involving dynamic processes such as oxidative stress, inflammatory cascades, extracellular matrix (ECM) metabolic disorders, and abnormal mechanical loading (Isa et al., 2023). During IVDD, a reduction in the number of NPCs is first observed, accompanied by an imbalance between the synthesis and degradation of the ECM (Sheyn et al., 2019). The significant decrease in aggrecan reduces the osmotic pressure of the disc matrix (Sha’ban et al., 2008). Concurrently, the decreased synthesis of type II collagen and increased synthesis of type I collagen reduce the elasticity and mechanical integrity of the IVD (Binch et al., 2021). These changes promote the infiltration of pro-inflammatory cytokines, serum proteins, and neurogenic mediators into the disc, triggering inflammation (Navone et al., 2017). In the late stage of degeneration, structural changes result in the loss of the biomechanical function of the IVD, with fissures forming in the AF, leading to protrusion of the NP. Simultaneously, sensory nerves within the AF and NP grow inward and develop vascularisation, which contributes to discogenic pain (Isa et al., 2023). The continued growth of bone spurs results in progressive narrowing of the intervertebral space, potentially leading to a significant loss of mobility (Stefanakis et al., 2012).

As the global population ages, the incidence of IVDD degeneration is increasing, placing greater demands on the development of effective treatments. Currently, the treatment methods for IVDD are mainly divided into conservative treatments and surgical treatments. Conservative treatment is primarily suitable for early-stage IVDD, with the primary goal of alleviating symptoms to improve quality of life. However, this treatment method is not practical in controlling the progression of IVDD and is therefore considered palliative care (Xin et al., 2022). Medication is the core method of conservative treatment, primarily including analgesics (such as non-steroidal anti-inflammatory drugs and opioid analgesics), muscle relaxants, corticosteroids, and so on (Lee et al., 2018). Although the medications above can relieve patients’ pain, they still have notable complications, such as the potential for addiction (Hale et al., 2005). Non-pharmacological conservative treatment methods can be implemented through bed rest, traction, exercise, and other approaches. These methods are non-invasive and low-cost. Although they cannot reverse the degenerative changes of IVDD, they can alleviate the pain caused by IVDD by repairing the microenvironment and preventing adhesions and re-injury (Altun and Yuksel, 2017). For patients with severe IVDD who have long suffered from pain, surgical treatment is the only viable option. The most prominent surgical technique is spinal fusion, which can be performed using minimally invasive or open surgical approaches, with access possible from the anterior, posterior, or posterolateral aspects. Studies have shown that spinal fusion can effectively relieve pain and improve mobility. However, in the long term, this surgical approach can lead to the loss of movement between adjacent vertebrae, increasing the load on surrounding tissues and the IVD, which may trigger IVDD in adjacent segments (Cannizzaro et al., 2022). Building on this, total disc replacement (TDA) offers an alternative surgical solution, as it provides vertebral mobility that spinal fusion surgery cannot. Long-term follow-up indicates that TDA does not increase the risk of degeneration in adjacent segments and yields satisfactory outcomes in pain relief (Ding et al., 2017). However, the high cost and concerns about complications prevent surgery from being the first choice for patients; surgery is often selected only when necessary.

The limitations of current options, ranging from traditional conservative methods to surgical interventions, have led researchers to seek new therapeutic alternatives (Pang et al., 2020). In recent years, the use of biomaterials has become a key area of research in biomedical science, especially for IVD regeneration therapies. These materials can act as scaffolds, offering mechanical support and aiding in the delivery of drugs or cells to enhance the biological function of the IVD (Mohd Isa et al., 2022). Hydrogels have garnered significant attention for their outstanding biocompatibility and adjustable drug-release properties. Many researchers are exploring the incorporation of bioactive factors to enhance the affinity of biomaterials for cells and promote cellular growth (Xing et al., 2021). At the same time, the emergence of stem cell technology has brought new hope for the treatment of IVDD, particularly with mesenchymal stem cells (MSCs), which have demonstrated positive effects in disc regeneration due to their excellent proliferation potential and differentiation capabilities (Richardson et al., 2016). Cell therapy has become a popular area of research, with findings indicating that stem cells can not only differentiate into disc cells to repair damage but also regulate the microenvironment by secreting bioactive factors, exhibiting multifunctional properties such as anti-inflammatory effects and promoting tissue regeneration (Bhujel et al., 2022).

This article provides a comprehensive review of recent advancements in IVDD research, with a focus on the development and application of biomaterials. By integrating findings from basic research, cutting-edge technologies, and clinical applications, we will systematically explore the pathological mechanisms of IVDD and its treatment. Special emphasis will be placed on applying emerging technologies such as novel biomaterials, cell therapy, and 3D printing in disc regeneration.

## 2 The pathological mechanisms of IVDD and the key role of oxidative stress

The pathological process of IVDD is a cascade reaction triggered by an imbalance in redox homeostasis, with oxidative stress playing a central driving role. The excessive accumulation of reactive oxygen species (ROS) in degenerated discs reaches concentrations 3 to 5 times higher than those in normal tissues, arising from multiple sources: approximately 60% of superoxide is contributed by mitochondrial electron transport chain leakage. Nicotinamide adenine dinucleotide phosphate (NADPH) oxidase recombinant NADPH oxidase 4 (NOX4) generates about 30% of hydrogen peroxide. The remaining ROS is produced by lipoxygenases and the Fenton reaction (Zhang et al., 2025a). This overload of ROS directly causes lipid peroxidation of the mitochondrial membranes, leading to the persistent opening of the inner membrane permeability transition pores and resulting in a decline in adenosine triphosphate (ATP) synthesis efficiency (Song et al., 2023). At the molecular level, ROS exacerbates the degradation of the ECM through dual pathways. On the one hand, it activates the nuclear transcription factor-κB (NF-κB) pathway, promoting the expression of pro-inflammatory factors, such as tumour necrosis factor-α (TNF-α) and interleukin 6 (IL-6) (Pa et al., 2023). On the other hand, ROS induces oxidative modifications of apoptosis signal-regulated kinase 1 (ASK1) in the mitogen-activated protein kinase (MAPK) pathway, which activates the c-Jun N-terminal kinase (JNK)/p38 signaling axis, resulting in enhanced transcription of matrix metalloproteinase-3 (MMP-3) and ADAMTS-5 (A disintegrin and metalloproteinase with thrombospondin motifs 5) (Shi et al., 2023a), which ultimately leads to an imbalance in the ECM synthesis/degradation ratio (Wang et al., 2024a). It is noteworthy that ferroptosis exhibits unique pathological characteristics during this process. The expression of Acyl-CoA synthetase long-chain family member 4 (ACSL4) is upregulated in the degenerated nucleus pulposus, increasing the polyunsaturated fatty acids-phospholipid it catalyses. Meanwhile, polyhydroxylated fullerenes reduce the Fenton reaction rate by 83% through chelation of Fe^2+^, resulting in the viability of nucleus pulposus cells recovering from 41% to 78% (Qin et al., 2025).

Mitochondrial dysfunction constitutes a core mechanism of energy metabolism imbalance, characterised by decreased activity of mitochondrial complexes I and III and increased electron leakage (Qiu et al., 2024). In the early stages of degeneration, the upregulation of peroxisome proliferator-activated receptor gamma coactivator 1-alpha (PGC-1α) promotes mitochondrial biogenesis. However, the accumulation of ROS leads to increased dynamin-related protein 1 (Drp1)-mediated mitochondrial fragmentation, resulting in decreased ATP production (Ji et al., 2025). Mitochondrial DNA (mtDNA) damage activates M1 macrophage infiltration via the cyclic GMP-AMP synthase-stimulator of interferon genes (cGAS-STING) pathway. In contrast, mitochondrial transcription factor A (TFAM) gene therapy can restore mtDNA integrity. The inflammation-oxidative stress positive feedback loop is self-amplified through the TLR4/MyD88/NF-κB axis, with the proportion of TLR4+ cells in degenerated intervertebral discs reaching 61%. Furthermore, ROS activates the NOD-like receptor family pyrin domain-containing 3 (NLRP3) inflammasome, resulting in increased levels of IL-1β and IL-18 (Wang et al., 2024b; Ma et al., 2025).

Dysregulation of epigenetic regulation exacerbates oxidative stress, as evidenced by increased methylation of the nuclear factor erythroid 2-related factor 2 (Nrf2) promoter, resulting in reduced Nrf2 expression. Additionally, the upregulation of histone deacetylase 3 (HDAC3) inhibits the activity of superoxide dismutase 2 (SOD2) (Zhang et al., 2025a; Hu et al., 2024a). In the disturbed non-coding RNA network, elevated levels of miR-34a-5p suppress sirtuin 1 (SIRT1) protein, while circ_0022383 reduces malondialdehyde (MDA) levels by adsorbing miR-125b-5p (Jiang et al., 2020). Biomechanical abnormalities (compression load > 1 MPa) induce Ca^2+^ influx through the (piezo-type mechanosensitive ion channel component 1) Piezo1 channel, activating NOX4 expression. Meanwhile, periodic stretching enhances the rate of ROS generation via the integrin β1-focal adhesion kinase (FAK) pathway (Li et al., 2025a).

**FIGURE 1:**
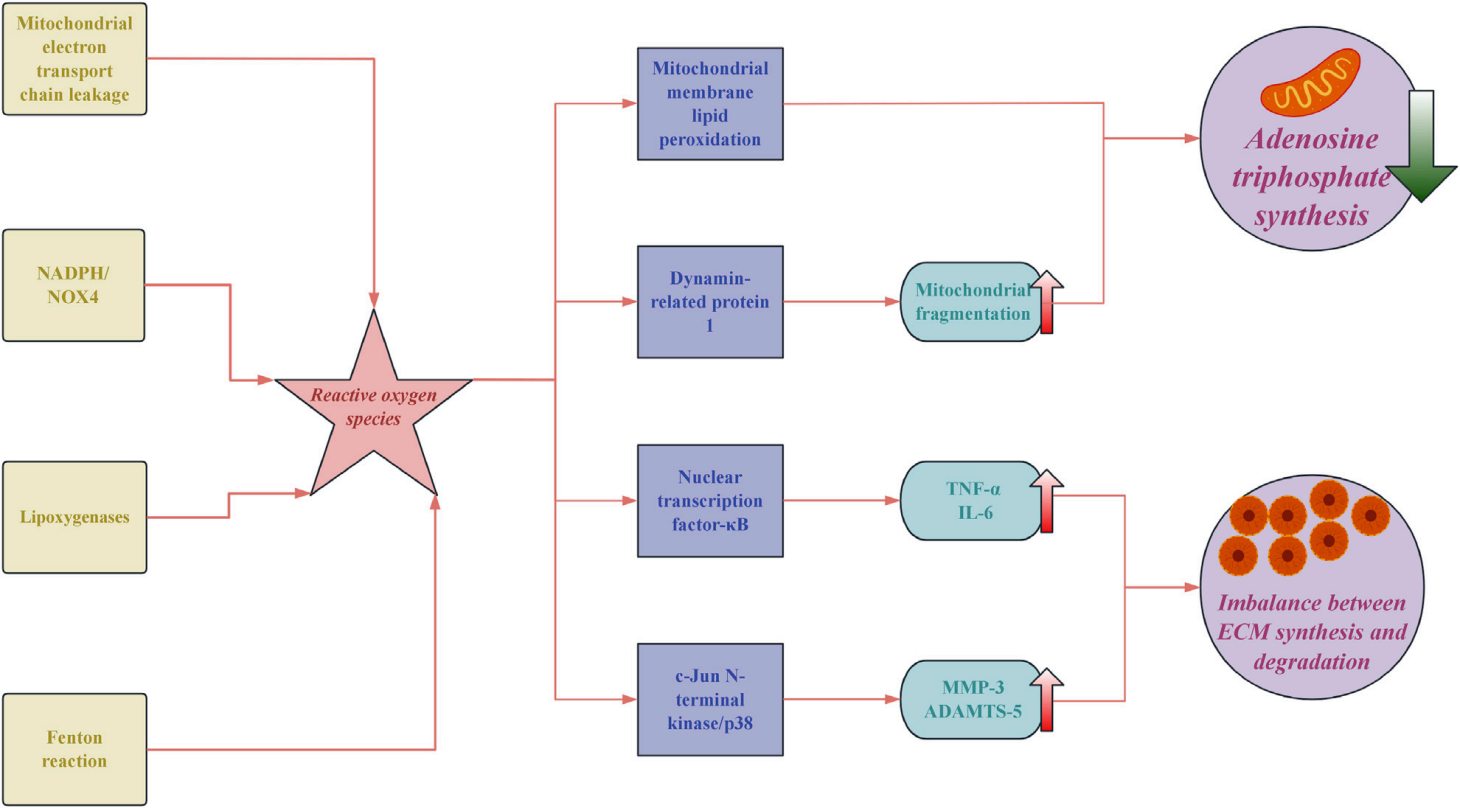
The core pathological mechanisms of oxidative stress in intervertebral disc degeneration. NADPH: nicotinamide adenine dinucleotide phosphate; NOX4: NADPH oxidase 4; TNF-α: tumor necrosis factor-α; IL-6: interleukin 6; MMP-3: matrix metalloproteinase-3; ADAMTSA-5: a disintegrin and metalloproteinase with thrombospondin motifs 5; ECM: extracellular matrix.

The pathological progression of IVDD) is driven by oxidative stress due to redox imbalance, with ROS levels in degenerated discs significantly elevated. Mitochondrial dysfunction plays a crucial role, as increased ROS impairs ATP synthesis and promotes inflammation via the NF-κB and MAPK pathways, leading to ECM degradation. Ferroptosis and epigenetic dysregulation further exacerbate oxidative stress, influencing cellular survival and function (Figure 1). Mechanical loading impacts ROS generation, creating a feedback loop that enhances degeneration. These findings highlight the need for multifaceted therapeutic approaches targeting oxidative stress, inflammation, and mechanical factors to combat IVDD effectively.

## 3 The core driving effect of oxidative stress damage

In the pathological process of IVDD, oxidative stress creates a cascaded pathological signaling network due to the imbalance between the excessive production and clearance of ROS. This imbalance directly leads to mitochondrial dysfunction and ferroptosis in NPCs (Mai et al., 2025). Mechanical stress, hypoxia, and the inflammatory microenvironment synergistically activate mitochondrial complexes I and III, as well as NOX, leading to elevated ROS levels in degenerated intervertebral discs compared to normal tissues. This increase in ROS activates the NF-κB pathway by oxidatively modifying I-kappa-B-kinase beta (IKKβ), inducing the release of IL-1β and TNF-α. Concurrently, the upregulation of the c-Fos/c-Jun complex promotes the expression of MMP-1 and MMP-3, resulting in an increased degradation rate of collagen type II and aggrecan (Wang et al., 2024b). When the concentration of H_2_O_2_ exceeds 50 μM, the activity of MMP-13 exhibits an exponential increase, revealing the critical threshold for antioxidant intervention. Ferroptosis, a new paradigm of programmed cell death, is characterised by decreased expression of GPX4 and elevated levels of lipid peroxidation products, such as MDA, in IVDD. Furthermore, HIF-1α inhibits ferritin-heavy polypeptide 1 (FTH1) through miR-210-3p, accumulating Fe^2+^, which subsequently generates OH through the Fenton reaction (Qin et al., 2025).

The interaction between oxidative stress and epigenetic regulation is manifested by the upregulation of ROS, which increases the expression of DNA methyltransferase 3B (DNMT3B), leading to elevated methylation levels of the SRY-related HMG-box gene 9 (Sox9) promoter and inhibiting the synthesis of aggrecan. However, the HDAC inhibitor suberoylanilide hydroxamic acid (SAHA) can reverse this process (Liu et al., 2024b). In the context of mitochondrial-lysosomal axis dysfunction, oxidative damage to mtDNA activates the cGAS-STING pathway, which inhibits lysosomal-associated membrane protein 1 (LAMP1) and causes the lysosomal pH to rise from 4.5 to 5.8. This increase leads to the release of cathepsin B. However, the mitochondrial-targeted antioxidant MitoQ can restore the pH to 4.9, resulting in a 2.3-fold increase in cell viability (Zhou et al., 2024a). Research on microenvironment remodeling indicates that periodic compressive stress elevates ROS levels in NPCs, activating transient receptor potential cation channel subfamily V member 4 (TRPV4)/Piezo1 channels. Inhibition of TRPV4 can reduce the secretion of IL-6 (Easson et al., 2023). In addition to the mechanisms previously described, oxidative stress significantly contributes to the apoptosis of NPCs and the progression of IVDD. Increased levels of ROS activate apoptotic pathways, notably the mitochondrial pathway, leading to the activation of caspase-3 and paracaspase (Li et al., 2022a). This cellular apoptosis is often associated with a decrease in the expression of anti-apoptotic proteins, such as Bcl-2, thereby increasing the vulnerability of NPCs to oxidative damage (Lin et al., 2021). The loss of NPCs not only reduces the biosynthetic capacity of the disc matrix but also increases mechanical stress on nearby cells, creating a cycle of degeneration.

Furthermore, oxidative stress impairs anabolic signaling, essential for maintaining extracellular matrix integrity. For instance, oxidative stress can inhibit TGF-β pathways, which are critical for producing extracellular matrix proteins such as aggrecan and type II collagen, necessary for disc hydration and structural integrity (Wang, 2020). Under normal physiological conditions, TGF-β stimulates the synthesis of critical factors such as Sox9, which plays a key role in maintaining the health and functionality of intervertebral discs. However, the dysregulation of this pathway due to oxidative stress can lead to a compromised ability to produce and maintain the disc matrix (Tsingas et al., 2020). Recent studies have highlighted the intricate relationship between gut microbiota and systemic oxidative stress, which can affect the health of individuals with IVD. Dysbiosis, or microbial imbalance, may exacerbate systemic inflammation and oxidative stress, thereby worsening IVDD (Zheng et al., 2024).

Oxidative stress plays a pivotal role in the pathological processes of IVDD, inducing mitochondrial dysfunction and ferroptosis in NPCs due to ROS imbalance. It activates pro-inflammatory pathways, accelerating the degradation of extracellular matrix components and promoting the apoptosis of NPCs, thereby exacerbating degeneration. Furthermore, oxidative stress disrupts TGF-β signaling, hindering matrix production. The gut microbiota’s influence on systemic oxidative stress adds complexity to IVDD. These findings underscore the importance of targeted therapies that address oxidative stress, inflammatory pathways, and microbial health to mitigate degeneration.

## 4 Mitochondrial dysfunction and energy metabolism imbalance

As the largest avascular tissue in the human body, IVD has energy metabolic characteristics closely associated with degeneration. Due to the passive diffusion of nutrients through the endplate, NPCs exist in a prolonged state of hypoxia and low glucose availability, forcing the cells to rely on glycolysis for energy production (Hartman et al., 2018). However, during the degeneration process, abnormal metabolic shifts can trigger a vicious cycle: compensatory enhancement of mitochondrial oxidative phosphorylation leads to an excessive generation of ROS. At the same time, the efficiency of glycolysis decreases, leading to a reduced ATP synthesis rate (Patila et al., 2019). The decreased stability of hypoxia-inducible factor HIF-1α further weakens glycolytic capacity, forcing cells to rely on a defective mitochondrial respiratory chain. During this process, an imbalance in mitochondrial dynamics becomes a core mechanism. In NPCs from patients with IVDD, there is an upregulation of the mitochondrial fission protein DRP1 and a downregulation of the fusion protein mitofusin 2 (MFN2), resulting in a significant increase in the proportion of fragmented mitochondria (Lin et al., 2023). The collapse of the membrane potential leads to the sustained opening of the mitochondrial permeability transition pore (mPTP), resulting in calcium efflux and a decreased calcium uptake capacity. This process activates the calpain-caspase12 apoptotic pathway.

Furthermore, the accumulation of succinate due to impaired citric acid cycle activity increases the ROS peak at Complex I through reverse electron transfer (RET), resulting in a reduction in mtDNA copy number and an elevated mutation rate of genes encoding Complexes I and IIII (Wang et al., 2024c). Dysregulation of the mitochondrial quality control (MQC) system further exacerbates the pathology. In an aging mouse model, the activity of the PINK1/Parkin pathway shows a stepwise decline with age, with Parkin protein expression in 12-month-old individuals being only 30% of that in 3-month-old mice, leading to a 65% reduction in the clearance rate of damaged mitochondria (Zhou et al., 2024a). Additionally, heightened sensitivity to ferroptosis is indicated by a 60% reduction in levels of the lipid peroxidation marker MDA upon intervention with the iron chelator deferoxamine (DFO) (Qin et al., 2025).

It can be confirmed that mitochondria are the primary targets of ROS. When ROS accumulation exceeds the capacity of antioxidant defences, it induces oxidative stress, leading to changes in mitochondrial morphology and function (Reed et al., 2014). Mitochondrial dynamics primarily include two major processes: fission and fusion. These dynamic changes ensure the mitochondria’s functionality in response to cellular demands (Zemirli et al., 2018). In the pathological process of IVDD, the balance between mitochondrial fusion and fission is disrupted, leading to abnormalities in the shape, size, and number of mitochondria. Most importantly, this imbalance affects mitochondrial quality, ultimately impacting cellular energy metabolism (Wang et al., 2022). Additionally, mitochondrial autophagy occurs through three mechanisms: (i) PINK1-Parkin-mediated, (ii) Parkin-dependent, and (iii) protein-mediated (Palikaras et al., 2018). In the context of IVDD, elevated levels of TNF induce the upregulation of Parkin in NPCs, while also increasing the expression of MFN2, resulting in excessive mitochondrial autophagy. It's accelerated aging ultimately results in the death of both NPCs and AF cells (Xu et al., 2019).

**FIGURE 2:**
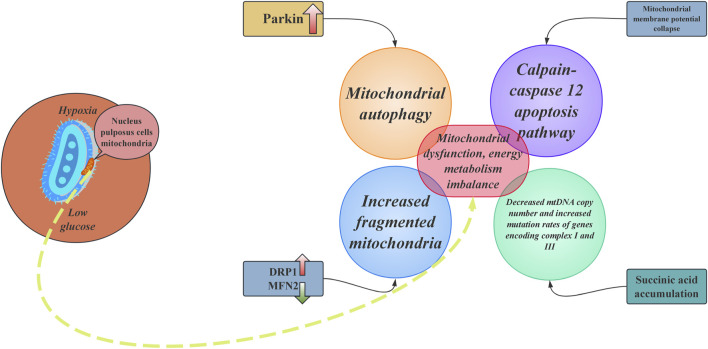
The core mechanisms of mitochondrial dysfunction and energy metabolism imbalance in nucleus pulposus cells. DRP1: dynamin-related protein 1; MFN2: mitofusin 2.

IVD exhibit unique energy metabolism due to hypoxia and nutrient limitations, primarily relying on glycolysis for ATP production. Metabolic changes during degeneration increase ROS production and impair mitochondrial function, causing mitochondrial fragmentation and apoptosis in NPCs (Figure 2). This mitochondrial dysfunction disrupts energy metabolism and worsens IVDD pathology, underscoring the vital role of mitochondrial dynamics in disc health and degeneration.

## 5 Development progress and application potential of antioxidant biomaterialse

These advanced materials are designed to scavenge ROS while also offering additional therapeutic benefits, including supporting cellular repair, enhancing bioactivity, and promoting tissue regeneration. In the field of nanoenzymes, carbon-based materials have attracted significant attention due to their biomimetic antioxidant and anti-inflammatory synergistic effects (Mai et al., 2025). In response to this pathological process, research on antioxidant biomaterials has shifted from solely targeting ROS clearance to multi-modal synergistic treatments. It includes innovative systems such as nanoenzymes, antioxidant nanoparticles, and multifunctional hydrogels. These advanced materials are designed to scavenge ROS while also offering additional therapeutic benefits, including supporting cellular repair, enhancing bioactivity, and promoting tissue regeneration. In the field of nanoenzymes, carbon-based materials have garnered significant attention due to their biomimetic antioxidant and anti-inflammatory synergistic effects. For example, research by Bu et al. developed glutathione-functionalized carbon dots (GSH-CDs), which form a 5 nm spherical structure through covalent bonding. These carbon dots exhibit GPx-like activity, reaching 82% of that of natural enzymes, and maintain stability for 72 h in an acidic microenvironment (pH 6.5–6.8).

Furthermore, GSH-CDs can reduce H_2_O_2_-induced ROS levels by 68% while upregulating the expression of SOD2 and HO-1 through the activation of the Nrf2-Keap1 pathway (Bu et al., 2024). In contrast, metal oxide nanoparticles enhance therapeutic effects through catalytic action and magnetic hyperthermia. For instance, Fe_3_O_4_@PDA nanoparticles reported by Zhu et al. exhibit a Fenton-like reaction catalytic efficiency of 7.8 times that of free Fe^2+^. When combined with alternating magnetic field (AMF) treatment, these nanoparticles can restore the MRI T2 signal of rat intervertebral discs to 82% of that observed in the normal group (Zhu et al., 2024). Rare earth-based materials, such as the CeO2@PEG nanoparticles designed by Yang et al., leverage the reversible valence state transition between Ce^3+^ and Ce^4+^ to achieve an IC50 for O_2_^−^ scavenging as low as 0.8 μg/mL. Furthermore, a single injection of these nanoparticles in a crab-eating macaque model can maintain the disc height index (DHI) at 89% of baseline values, highlighting their long-lasting antioxidant effects and potential for ECM regeneration (Yang et al., 2025).

The multifunctional hydrogel system has achieved a breakthrough in antioxidant efficacy through a three-dimensional network structure and intelligent, responsive design. The nanocomposite hydrogel developed by Luo et al. possesses a storage modulus of 14.8 kPa and maintains a 1,1-Diphenyl-2-picrylhydrazyl radical scavenging rate of over 85% for 24 h. Furthermore, by the sustained release of TGF-β1, the proportion of positively stained regions for Collagen II synthesis was enhanced to 52% (Luo et al., 2025). Xu et al. developed a photopolymerized hydrogel by combining MnO2 nanosheets with GelMA, which reduced the H_2_O_2_ concentration from 10 mM to 1.2 mM within 5 min. This intervention improved the Pfirrmann score of the rat IVDD model from 4.2 to 2.8 (61). Li et al. (2025) developed a chitosan/alginate hydrogel (CS/SA@Exo) that, through the sustained release of exosomes, reduced the apoptosis rate of NPCs from 41% to 15%. Additionally, in a pig model, it restored the IVD water content to 78% of that in the normal group (Li et al., 2025b).

Despite significant advancements in laboratory research, clinical translation still faces challenges related to long-term safety (such as hepatotoxicity from metallic nanoparticles), precise delivery (due to limitations in anatomic permeability), and standardised evaluation (Mai et al., 2025). Polyphenolic compounds have natural antioxidant properties that can scavenge ROS, reduce inflammation, and prevent oxidative stress in NPCs (Lai et al., 2019). As a representative of polyphenolic compounds, resveratrol can scavenge ROS and regulate mitochondrial dysfunction. This antioxidant capability reduces apoptosis in NPCs, prevents IVDD, and promotes regeneration (Jiang et al., 2018). The related mechanisms involve the NF-κB pathway, the JAK/STAT3 pathway, and the AKT-FoxO1-SIRT1 axis (Wu et al., 2021; He et al., 2019). Furthermore, apart from NPCs, the antioxidant effects of resveratrol are also beneficial in AF cells, which have therapeutic implications for alleviating low back pain caused by IVDD (Shan et al., 2021). However, despite the advantages of high biocompatibility and low toxicity, polyphenolic compounds cannot avoid poor solubility and bioavailability. On this basis, researchers have utilised carriers (e.g., hydrogels or nanomaterials) to deliver them to the exact site of the IVDD (Wang et al., 2020). For example, nanoscale network components composed of copper ions and epigallocatechin gallate exhibit antioxidant, anti-apoptotic, and anti-inflammatory abilities, effectively removing ROS (Zhou et al., 2024b). Wang et al. designed an injectable hydrogel encapsulated with tannic acid nanoparticles, which can eliminate intracellular ROS by downregulating TNF-α expression, thereby repairing nucleus pulposus cells and alleviating IVDD (Wang et al., 2023b).

The emergence of antioxidant materials presents innovative approaches and solutions for addressing IVDD. These materials offer dual benefits: they protect disc cells from oxidative damage by mitigating oxidative stress and stabilising the ECM, while also reducing inflammation associated with oxidative stress, thus effectively alleviating pain and other discomforts (Mai et al., 2025). Among the various types of antioxidant materials, hydrogels are noted for their excellent biocompatibility and enhanced pharmacokinetic properties (Li et al., 2023). Meanwhile, nanomaterials provide extensive antioxidant coverage and enhance the disc’s inherent repair mechanisms (Zhu et al., 2022). Polyphenols stand out due to their low cost of extraction, safety, and good tolerability. However, despite these advantages, uncertainties remain about the long-term efficacy of all antioxidant biomaterials, and their safety and potential side effects have yet to be thoroughly assessed and validated. Consequently, most current research is confined to preclinical investigations (Song et al., 2021). Future directions include the development of biomimetic gradient antioxidant scaffolds, ROS-responsive gene editing materials, and the application of artificial intelligence to antioxidant design. In summary, antioxidant biomaterials offer a novel approach to treating IVD degeneration through multifaceted regulatory mechanisms. However, their clinical application still requires interdisciplinary collaboration to overcome technical bottlenecks related to material biocompatibility, targeted delivery, and scalable production.

## 6 Breakthroughs in nanoenzymes and antioxidant nanoparticles

Nanoenzymes, a class of nanomaterials with enzyme-like catalytic activity, mimic the functions of natural antioxidant enzymes. In the antioxidant treatment of IVDD, they demonstrate efficient ROS scavenging capabilities, spatiotemporally controlled drug delivery characteristics, and multivalent synergistic effects. Moreover, their stability and environmental adaptability are significantly superior to those of traditional antioxidants (Yu et al., 2020). In biomimetic design, the glutathione-modified carbon quantum dots developed by Bu et al. achieve a GPx catalytic efficiency of 80% compared to natural enzymes by regulating surface functional groups. Additionally, they reduce the levels of ROS in NPCs and target the inhibition of cellular senescence mediated by the p16/p21 pathway (Bu et al., 2024). Shi et al. developed a core-shell structured nanoenzyme using co-doped NiO nanoparticles (CNO) as the core, coated with polydopamine. This nanoenzyme effectively scavenges various oxygen-free radicals, protects NPCs, and inhibits the progression of IVDD (Shi et al., 2023b). In metal oxide nanoparticles, cerium oxide nanoparticles utilise the Ce^3+^/Ce^4+^ redox cycle to influence extracellular matrix and collagen formation by regulating the PI3K-Akt and cell cycle signaling pathways, promoting osteogenic differentiation and mineralisation (Li et al., 2025c). Wang et al. constructed core-shell structured nanoenzymes with co-doped NiO nanoparticles as the core and a polydopamine shell, which exhibit various antioxidant enzyme-like activities. By reducing intracellular ROS in NPCs to H_2_O and O_2_, these nanoenzymes protect NPCs from stunted proliferation, abnormal metabolism, and inflammation, thereby restoring ECM homeostasis (Wang et al., 2024d).

Iron-based nanoparticles (IONPs) break through the limitations of single therapies through multifunctional modulation. Zhu et al. confirmed that IONPs can switch CAT/GPx activity based on H_2_O_2_ concentration (Zhu et al., 2024). The MnO2/GelMA composite hydrogel induces the expression of antioxidants and autophagy through the SIRT1/NRF2 pathway, effectively scavenging intracellular ROS and producing an antioxidative effect, thereby improving the microenvironment of IVD (Xu et al., 2024). A novel carbon dot nanoenzyme (N-acetylcysteine-derived carbon dots) exhibits superoxide dismutase, catalase, and glutathione peroxidase-like activities, as well as total antioxidant capacity. It can scavenge free radicals, maintain mitochondrial balance, and inhibit cellular aging, thus reducing the expression of inflammatory factors in NPCs, which offers a potential clinical strategy for managing IVDD (Wu et al., 2023). Utilizing a cationic polymer brush-coated carbon nanotube (oCNT-pb) as a siRNA delivery platform, LINC02569 siRNA (si-02569) is targeted and transported to NPCs, which effectively alleviates the inflammatory response in NPCs by inhibiting P65 phosphorylation and preventing its translocation to the nucleus, while also mitigating cellular senescence by reducing P21 expression (Huang et al., 2022). Polydopamine nanoparticles mitigate intervertebral disc degeneration by antagonizing ferroptosis in NPCs through the scavenging of ROS, chelation of iron ions, ubiquitination of GPX4, and downregulation of malondialdehyde and lipid peroxide production (Yang et al., 2023). The carbonised Mn-containing nanodots developed by Sun et al. demonstrate a stronger ROS scavenging ability and ECM remodeling capacity than the classic antioxidant N-acetylcysteine, by inhibiting necroptosis in NPCs, thereby providing an effective intervention in a rat model of IVDD (Sun et al., 2024). Selenium nanoparticles can inhibit the expression of matrix-degrading enzymes, upregulate the expression of proteoglycan and type II collagen, and maintain redox homeostasis by activating glutathione peroxidase 1 (GPX1). This process helps preserve mitochondrial integrity and restore impaired mitochondrial energy metabolism, thereby retaining hydration in the nucleus pulposus tissue, promoting matrix deposition, and effectively alleviating the progression of IVDD (He et al., 2024).

Prussian blue nanoparticles enhance mitochondrial structure and improve antioxidant capacity by promoting the expression of mRNA and proteins related to the redox enzyme system, as well as stabilising SOD1 against ubiquitination and proteasomal degradation, thereby rescuing IVDD induced by ROS (Zhou et al., 2022). Fullerene nanoparticles have been shown to possess excellent ROS scavenging capabilities. In an IVDD animal model, the intra-discal injection of fullerenes significantly reduced H_2_O_2_-induced cytotoxicity and cellular ROS levels, increased water and proteoglycan content. It suppressed ectopic bone formation, thereby preventing IVDD (Yang et al., 2014).

Nanoenzymes, which mimic natural antioxidant enzymes, offer promising therapeutic potential for IVDD by efficiently scavenging ROS and enabling controlled drug delivery. Various studies have demonstrated their ability to protect NPCs, enhance extracellular matrix formation, and promote cellular homeostasis through multiple pathways. Innovative strategies, such as iron-based and Prussian blue nanoparticles, further enhance antioxidant capacities and improve mitochondrial integrity. Overall, the diverse mechanisms of these nanomaterials underscore their potential as multifunctional agents in managing oxidative stress and inflammation in IVDD, highlighting the need for ongoing research to optimise their clinical applications.

## 7 Synergistic antioxidant strategies of multifunctional hydrogels

Multifunctional hydrogels employ synergistic antioxidant strategies to address the limitations of traditional antioxidants through various technological advancements. The core of this approach lies in the integration of precise drug delivery, dynamic mechanical adaptability, and microenvironment-responsive mechanisms. The results of a meta-analysis of preclinical animal studies indicate that exosome-loaded hydrogels significantly improve the IVD height index score and reduce magnetic resonance imaging grading by inhibiting ECM degradation and alleviating cellular senescence and degeneration, demonstrating potential beyond enhancing the structure and function of the IVD (Wang et al., 2025). Regarding the synergistic enhancement of antioxidant activity and drug delivery, the nano-composite hydrogel utilises the Pickering emulsion method to load quercetin nanoparticles precisely. The ROS-triggered temporal release is accomplished through π-π stacking effects. When combined with TGF-β1, this promotes ECM synthesis, thereby matching the compressive modulus to the mechanical requirements of the natural nucleus pulposus (Luo et al., 2025). The MnO2/GelMA system activates the SIRT1/NRF2 pathway through a cascade reaction involving nanoenzyme-catalysed decomposition of H_2_O_2_ and the regeneration cycle of α-lipoic acid, restoring the intervertebral disc O_2_ partial pressure to normal levels in a crab-eating macaque model (Xu et al., 2024). The “sandwich” structured hydrogel further innovates the temporal release system, with the outer layer of SODm achieving rapid ROS scavenging (70% release within 24 h), while the inner layer, which is MMP-2 responsive, releases TGF-β1 in a delayed manner, peaking at 48 h, which significantly enhances the expression of aggregated proteoglycans (Yang et al., 2024). Innovative breakthroughs in hydrogel materials within the biomedical field are focused on three key dimensions: optimisation of self-healing properties, intelligent and responsive design, and integration of multimodal functionalities. Self-healing hydrogels achieve a breakthrough in mechanical properties through dynamic bond restructuring, addressing the mechanical compatibility requirements for intervertebral disc repair. The hydrogen bond-based PVA-boric acid system exhibits a tensile elongation of 800% and a self-healing efficiency greater than 90% (Guo et al., 2025). The pathological microenvironment of IVDD exhibits high heterogeneity, characterized by local pH reduction (pH 6.2–6.8), overload of ROS (3-5 times higher than in normal tissue), and enhanced activity of matrix-degrading enzymes such as MMP-3 and ADAMTS-5. Smart, responsive hydrogels have demonstrated the ability to sense changes in this microenvironment, triggering the release of drugs or structural remodeling. This technology signifies a shift from a passive filling approach to an active regulatory treatment paradigm. To achieve precise regulation of the acidic microenvironment (pH < 6.5), borate ester-bonded EGCG hydrogels demonstrate an impressive cumulative drug release rate of 85% over 24 h at pH 6.5, 2.3 times higher than that in neutral conditions. The released EGCG downregulates the expression of IL-1β and TNF-α by inhibiting the nuclear translocation of NF-κB.

Additionally, hyaluronic acid modified with α-lipoic acid (HA-LA) imparts self-healing capabilities to the hydrogel, thereby enhancing its potential for therapeutic applications in the context of IVDD (Liu et al., 2024b). In response to the oxidative stress cascade triggered by ROS overload, the advanced PVA-tsPBA@SLC7A11 system utilizes phenylboronate ester bonds to release SLC7A11 mRNA in the presence of H_2_O_2_. This induction significantly reduces iron death markers, including PTGS2 and ACSL4, in NPCs by up to 70%. Additionally, the outer layer of HAMA (hyaluronic acid modified with α-lipoic acid) forms a ROS gradient-responsive barrier, effectively balancing therapeutic release with mechanical support, thereby enhancing the overall therapeutic efficacy in IVD (Gao et al., 2024). Wang et al. developed an anti-aging hydrogel by conjugating phenylboronate-modified gelatin methacryloyl with quercetin. This hydrogel effectively reduced the expression of aging markers in NPCs. It restored metabolic homeostasis, helping to maintain intervertebral disc height and NP tissue structure, while significantly alleviating sensitivity to mechanical pain (Wang et al., 2024e). Based on autophagy, Song et al. developed a multifunctional DNA hydrogel loaded with miR-5590. By administering it via local injection, miR-5590 is continuously released to induce autophagy in NPCs, subsequently inhibiting cell apoptosis and regulating the metabolic balance within the ECM, thereby hindering the progression of IVDD (Qingxin et al., 2023). Similarly, a high-strength biopolymer hydrogel based on zinc-oxidized sodium alginate-gelatin demonstrated good biocompatibility after loading with antagomir-204-3p. In addition to crosslinking with nucleus pulposus tissue to form a high-strength collagen network and improving the mechanical properties of the IVD, it also plays a role in maintaining the metabolic balance of the ECM, thereby restoring the height of the IVD, retaining its moisture, and maintaining the structural integrity of the tissue (Chen et al., 2023).

Recent advancements in multifunctional hydrogels are revolutionizing antioxidant therapies for IVDD through innovative strategies, including precise drug delivery, dynamic adaptability, and microenvironment responsiveness. These hydrogels enhance antioxidant activity, promote extracellular matrix synthesis, and effectively respond to pathological conditions. The MnO2/GelMA and borate ester-bonded EGCG hydrogels demonstrate significant potential in regulating oxidative stress and inflammatory responses, thereby preserving disc integrity and functionality. Incorporating self-healing properties and smart responsiveness optimizes mechanical compatibility and therapeutic efficacy.

## 8 Synergistic innovations in organisational engineering and stem cell therapy

The field of IVDD treatment is undergoing a revolutionary shift from traditional surgery to regenerative medicine, where the interdisciplinary integration of tissue engineering and stem cell therapy offers innovative pathways for regenerating the complex three-dimensional structure of the disc. A meta-analysis comprising eight randomised controlled trials (RCTs) and eight observational studies demonstrates that regenerative medicine therapies for IVD (including MSCs and platelet-rich plasma) can effectively treat patients with disc-related low back pain, with a quality of evidence rating of level III (Manchikanti et al., 2024). The results of a meta-analysis involving 22 studies indicate that the inhibition of stem cells in the IVD of quadrupedal animals slows down the occurrence of IVDD, which is manifested explicitly as an increase in the IVD height index, an increase in the expression of type II collagen mRNA, and a decrease in histological grading of disc degeneration (Wang et al., 2015). The acellular therapeutic paradigm of stem cell extracellular vesicles (EVs) has achieved breakthroughs in targeted delivery through engineered modifications. The Gel@EXski + scFv system developed by Li et al. enhances the targeting efficiency of EVs in a crab-eating macaque model by 3.2 times by employing surface-anchored single-chain variable antibodies (scFv). Furthermore, this system successfully reverses the TGF-β/Smad3 pathway by carrying ski mRNA, thereby restoring the collagen I/II ratio from 5.8:1 to 1.3:1 (62). In addition, mesenchymal stem cell-derived small extracellular vesicles exert their anti-aging effects by delivering miR-105-5p to aging NPCs, downregulating the levels of cAMP-specific phosphodiesterase PDE4D and activating the Sirt6 pathway. This novel acellular therapeutic tool overcomes the limitations associated with the direct transfer of MSCs (Sun et al., 2021). The micelle delivery system loaded with coenzyme Q10 targets mitochondrial ROS, enhancing the viability of mesenchymal stem cells and restoring mitochondrial structure and function. This approach helps maintain intervertebral disc height in rats and alleviates IVDD (Sun et al., 2023). A linear structure microneedle designed to match the annulus fibrosus ring structure effectively adheres to the annulus fibrosus by loading extracellular vesicles derived from bone marrow mesenchymal stem cells. These vesicles encapsulate the microRNA (miRNA) 378, which regulates mitochondrial autophagy. This system can slowly release extracellular vesicles, ultimately preventing the progression of IVDD by restoring mitochondrial autophagy, promoting the proliferation and migration of AF cells, and inhibiting pathological remodeling of the ECM (Hu et al., 2024b). High-throughput sequencing analysis showed that the expression level of miR-125b-5p was lower in degenerated IVD tissue. MSC-derived exosomes can deliver miR-125b-5p to the target TRAF6, inhibiting NF-κB activation and acting as an apoptosis inhibitor in NPCs, thereby slowing the progression of IVDD (Duan et al., 2023). Through inhibiting inflammatory mediators and NLRP3 inflammasome activation, mesenchymal stem cell-derived exosomes play a crucial role in exerting antioxidative and anti-inflammatory effects (Xia et al., 2019). Further studies have shown that exosomes downregulate the NLRP3 inflammasome by delivering miR-302c, thereby alleviating pyroptosis in NPCs (Yu et al., 2023), which suggests that exosome therapy may be a promising treatment approach.

Stem cell therapy is considered a promising innovative treatment for IVDD. It relies on the differentiation capacity of stem cells to promote the growth of NPCs, restore the structural integrity of degenerated IVDs, and secrete anti-inflammatory cytokines to reduce the occurrence of inflammation, thereby reversing the degenerative process (Molladavoodi et al., 2020; Munda and Velnar, 2024). In the 1990s, researchers first attempted to implant autologous NPCs into degenerated IVD. The results showed that the implantation of NPCs delayed AF degeneration and had a protective effect on the remaining normal NPCs (Nishimura and Mochida, 1998). MSCs can differentiate into osteoblasts, adipocytes, or chondrocytes, and under specific conditions, they can even differentiate into cells resembling NPCs. Researchers are gradually realizing that MSCs are the most promising source of cells for IVD regeneration (Clarke et al., 2014; Schroeder et al., 2015). The consistency of *in vivo* and *in vitro* animal experiments emphasizes the potential of stem cell therapy for treating IVDD. In rabbit models, the injection of stem cells has been observed to lead to proliferation and differentiation into cell types with phenotypic characteristics of NPCs. These differentiated cells are capable of synthesizing type II collagen and proteoglycans, improving the ECM, and restoring IVD height (Sakai et al., 2005). In addition to their differentiation capabilities, a reduction in pro-inflammatory cytokines (IL-6, IL-8, TNF-α) has been observed following the injection of MSCs, indicating that MSCs secrete immunomodulatory factors through paracrine effects to create a favorable microenvironment (Miguelez-Rivera et al., 2018). However, it is essential to note that most current research is derived from preclinical studies, and caution should be exercised when translating these findings into clinical trials (Oehme et al., 2015). A few clinical studies appear to validate the therapeutic effects of MSCs, including a study involving 33 patients with chronic back pain, which showed an average 60% improvement in pain 3 years after injection of autologous bone marrow-derived MSCs (BM-MSCs). Additionally, a reduction in the size of herniated intervertebral discs was noted on MRI (Centeno et al., 2017). Randomised controlled trial results showed that, compared to the control group, the MSCs treatment group exhibited rapid and significant improvement in symptoms, whereas symptoms in the control group worsened (Noriega et al., 2017). A multicenter, randomised, controlled trial involving 100 patients demonstrated that the injection of allogeneic BM-MSCs significantly outperformed the control group in reducing pain and improving overall function (Amirdelfan et al., 2021). Although the results of clinical trials are encouraging, current research still has limitations, including a lack of control groups, insufficient sample sizes, unclear objective outcome measures, and short follow-up periods. Therefore, higher-quality studies are required to provide more definitive clinical evidence supporting the clinical potential of MSCs in treating IVDD. Additionally, conducting basic research to elucidate the exact molecular mechanisms underlying stem cell therapy is also essential (Xie et al., 2021).

The groundbreaking advancements in 3D bioprinting technology offer a precise solution for regenerating IVD gradient structures, with the core being the deep integration of multi-material collaborative printing and biomimetic structural design. In the field of multi-material printing technology, the team led by Tianpeng Xu successfully constructed a continuous gradient scaffold with a compressive modulus ranging from 0.5 MPa to 18 MPa by synchronously printing three types of bioinks using a “coaxial four-head” system. The orientation of the collagen fibers reached 82%, creating a gradient scaffold with a continuous transition of elastic modulus from the cartilage end to the bone end. This scaffold exhibited a threefold improvement in compressive fatigue life compared to traditional homogeneous scaffolds (Xu et al., 2025). Zhu et al. combined 3D printing and electrospinning to construct a composite scaffold for artificial IVD. Morphological and mechanical testing indicated that the structure and mechanical properties of the artificial IVD scaffold are similar to those of natural IVD. Animal experiments demonstrated that the biomimetic artificial IVD scaffold can maintain IVD integrity and remodel the ECM (Zhu et al., 2021). Liu et al. utilized electrohydrodynamic 3D printing technology to fabricate high-resolution polycaprolactone scaffolds that mimic the structural characteristics of natural AF. The results indicated that scaffold implantation helps maintain intervertebral disc height, reduces NP moisture loss, and partially restores the biomechanical function of the IVD (Liu et al., 2023a). Zhang et al. prepared a novel poly (glycolide-co-caprolactone)@polylactide (PLA)-b-aniline pentamer (AP)-b-PLA/chitosan-ϵ-polylysine (PGCL@1PAP/10CSPL) scaffold using 3D printing technology, which serves as an implant for the treatment of IVDD. *In vitro* and *in vivo* experiments demonstrated that this composite scaffold enhanced osteogenic differentiation, promoted angiogenesis, and accelerated abnormal NP degeneration, making it suitable for lumbar interbody fusion (Zhang et al., 2025b).

The approach to treating IVDD is evolving from conventional surgery to regenerative medicine, incorporating tissue engineering and stem cell therapy. Advances in stem cell-derived EVs are improving targeted drug delivery, while sophisticated 3D bioprinting techniques are producing biomimetic scaffolds that enhance mechanical properties. These innovations signal a promising shift in IVDD management, focusing on targeted and multifunctional therapeutic strategies.

## 9 Discussion

IVDD is a complex and multifaceted pathological condition that requires innovative treatment strategies targeting its underlying mechanisms. The shift from conventional therapies to regenerative medicine is not just a trend but a necessary response to the growing clinical burden of low back pain and disability caused by IVDD (Kirnaz et al., 2022). With the aging population, the need for effective treatments is increasing, driving researchers to investigate new methods that incorporate advances in biomaterials, tissue engineering, and stem cell therapy (Mohd Isa et al., 2022).

At the cellular level, IVDD is marked by oxidative stress, inflammation, and metabolic dysfunction, which significantly contribute to the degeneration of the IVD (8). Oxidative stress plays a critical role, as excessive production of ROS leads to mitochondrial dysfunction and apoptosis in NPCs. The signaling events triggered by ROS disrupt the balance of ECM synthesis and degradation, compromising the disc’s mechanical integrity, which highlights the need for therapies that target ROS and promote cellular health and function (Chen et al., 2024).

Recent advancements in biomaterials have demonstrated potential in mitigating the harmful effects of oxidative stress. For instance, multifunctional hydrogels and nanoenzymes are engineered to neutralize ROS while delivering bioactive agents that promote cellular proliferation and tissue regeneration (Jarrah et al., 2023). Hydrogels, in particular, offer significant benefits due to their customizable properties, which can be adjusted to replicate the natural disc environment (Bing et al., 2023). This not only provides mechanical support but also creates a favorable microenvironment for cell attachment and migration. The ability of these materials to adapt to physiological conditions is crucial for their effectiveness in clinical settings (Wang et al., 2024f).

The application of stem cell therapy represents another transformative approach in the treatment of IVDD. MSCs exhibit remarkable potential for differentiating into disc-like cells and for secreting bioactive factors that promote ECM synthesis and reduce inflammation (Bhujel et al., 2022). Recent advances in the use of EVs derived from MSCs have opened new avenues for therapy. EVs can be engineered for targeted delivery, enhancing their therapeutic efficacy while minimizing systemic side effects. Innovations such as the Gel@EXski + scFv system exemplify these advancements, showcasing the potential for improved targeting and delivery of therapeutic RNA to modulate degenerative pathways, like the TGF-β/Smad3 signaling (Hu et al., 2023). This approach not only restores the collagen I/II ratio critical for disc integrity but also holds promise for reversing the degenerative process.

The integration of advanced manufacturing technologies, such as 3D bioprinting, is transforming scaffold design for IVDD treatment. These technologies enable the creation of scaffolds with precise mechanical and biological properties, closely mimicking the complex anatomy of IVD (De Pieri et al., 2020). For example, gradient scaffolds that provide varying mechanical properties, ranging from cartilage to bone, can enhance the biomechanical function of implanted materials, thereby improving load distribution and reducing the risk of further degeneration. The success of these treatments hinges on their ability to ensure biomechanical stability, promote cellular activity, and integrate with surrounding tissues (Bowles and Setton, 2017).

However, substantial barriers remain to the clinical implementation of these innovative therapies. Challenges such as ensuring long-term safety, addressing biocompatibility, achieving precise delivery, and maintaining patient adherence demand interdisciplinary collaboration among researchers, clinicians, and materials scientists. For instance, while nanoparticles and EVs offer significant benefits, their interaction with biological systems may present unforeseen complications related to immune responses or toxicity (Liu et al., 2023b). Stem cell transplantation also poses several issues that need to be addressed, such as what is the safe number of cells that can be transplanted, whether there is a risk of immune response from the foreign cell transplantation, whether it may lead to increased pain, and whether there are any unforeseen ethical issues (Taylor and Erwin, 2024). Although current studies support the notion that stem cell transplantation exerts therapeutic effects by secreting immunomodulatory factors, growth factors, and anti-inflammatory cytokines, several limitations remain in the existing research, including insufficient sample sizes, selection bias, short follow-up periods, and a lack of control groups (Munda and Velnar, 2024; Zhang et al., 2022). Furthermore, rigorous preclinical and clinical testing will be essential to assess the efficacy and safety profiles of these emerging technologies.

The complexity of IVDD necessitates a multidisciplinary approach that encompasses not only the development of innovative treatments but also a deeper understanding of the disease pathology. Future directions may involve exploring the gut microbiome’s influence on systemic oxidative stress and IVDD, as emerging evidence suggests that dysbiosis can exacerbate oxidative damage and inflammatory responses (Li et al., 2022b). Understanding these relationships could lead to synergistic therapeutic strategies that combine traditional treatments with microbiome modulation, ultimately improving patient outcomes.

## 10 Conclusion

In conclusion, the advancement of regenerative medicine through the integration of biomaterials, stem cell therapy, and innovative manufacturing techniques holds great promise for the treatment of IVDD. By addressing the multifaceted nature of the disease and exploring the interplay between oxidative stress, inflammation, and metabolism, we can develop a comprehensive therapeutic paradigm that not only alleviates symptoms but also restores the functionality and integrity of the intervertebral disc. Continued research and clinical trials are essential in bridging the gap between laboratory developments and effective patient care.
