# Supplementary material for: Innovative strategies in combating intervertebral disc degeneration: pathological mechanisms and biomaterial advancements
Source: Front Bioeng Biotechnol. 2025 Aug 14;13:1643222. doi: 10.3389/fbioe.2025.1643222 (PMC12391112; doi:10.3389/fbioe.2025.1643222)
Supplement: Supplementary file 1 [file Supplementaryfile1.docx]

Supplementary Material

# Supplementary Figures


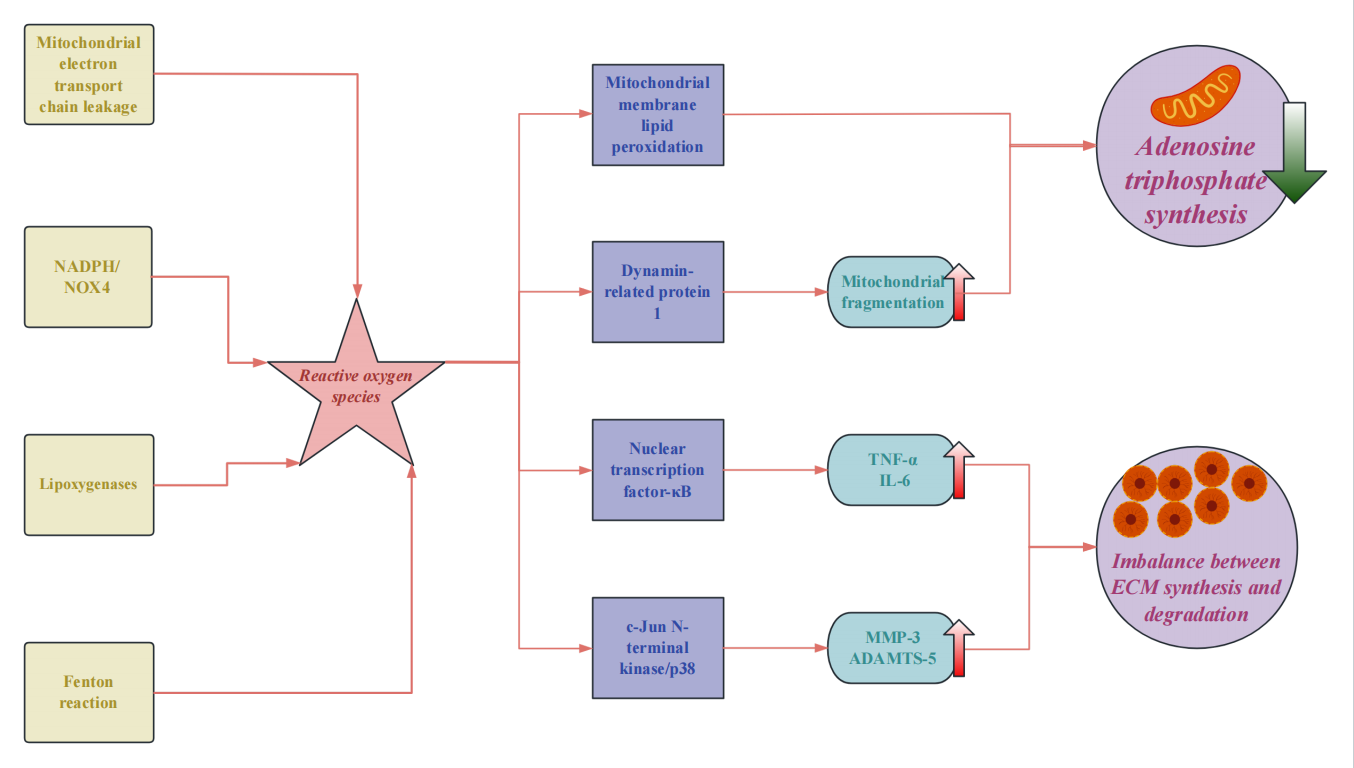


**Supplementary Figures 1.** The core pathological mechanisms of oxidative stress in intervertebral disc degeneration. NADPH: nicotinamide adenine dinucleotide phosphate; NOX4: NADPH oxidase 4; TNF-α: tumor necrosis factor-α; IL-6: interleukin 6; MMP-3: matrix metalloproteinase-3; ADAMTSA-5: a disintegrin and metalloproteinase with thrombospondin motifs 5; ECM: extracellular matrix.


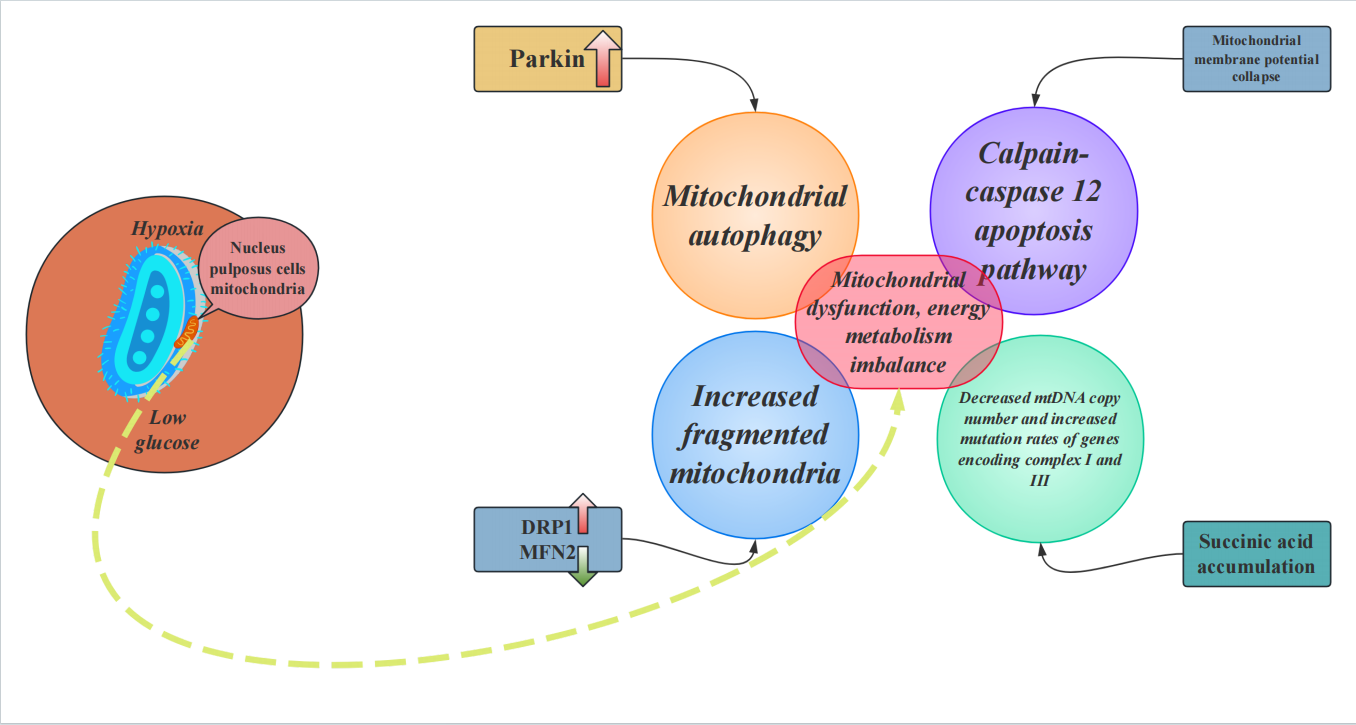


**Supplementary Figures 2.** The core mechanisms of mitochondrial dysfunction and energy metabolism imbalance in nucleus pulposus cells. DRP1: dynamin-related protein 1; MFN2: mitofusin 2.
